# Construction and validation of a prognostic model based on 11 lymph node metastasis‐related genes for overall survival in endometrial cancer

**DOI:** 10.1002/cam4.4844

**Published:** 2022-07-02

**Authors:** Hong Wu, Haiqin Feng, Xiaoli Miao, Jiancai Ma, Cairu Liu, Lina Zhang, Liping Yang

**Affiliations:** ^1^ Department of Obstetrics and Gynecology Handan Central Hospital Handan China

**Keywords:** endometrial cancer, HMGB3, lymph node metastasis, mutation, risk signature

## Abstract

**Background:**

Endometrial cancer (EC) is one of the most common malignant tumors in female reproductive system. The incidence of lymph node metastasis (LNM) is only about 10% in clinically suspected early‐stage EC patients. Discovering prognostic models and effective biomarkers for early diagnosis is important to reduce the mortality rate.

**Methods:**

A least absolute shrinkage and selection operator (LASSO) regression was conducted to identify the characteristic dimension decrease and distinguish porgnostic LNM related genes signature. Subsequently, a novel prognosis‐related nomogram was constructed to predict overall survival (OS). Survival analysis was carried out to explore the individual prognostic significance of the risk model and key gene was validated in vitro.

**Results:**

In total, 89 lymph node related genes (LRGs) were identified. Based on the LASSO Cox regression, 11 genes were selected for the development of a risk evaluation model. The Kaplan–Meier curve indicated that patients in the low‐risk group had considerably better OS (*p* = 3.583e−08). The area under the ROC curve (AUC) of this model was 0.718 at 5 years of OS. Then, we developed an OS‐associated nomogram that included the risk score and clinicopathological features. The concordance index of the nomogram was 0.769. The survival verification performed in three subgroups from the nomogram demonstrated the validity of the model. The AUC of the nomogram was 0.787 at 5 years OS. Proliferation and metastasis of HMGB3 were explored in EC cell line. External validation with 30 patients in our hospital showed that patients with low‐risk scores had a longer OS (*p*‐value = 0.03). Finally, we revealed that the most frequently mutated genes in the low‐risk and high‐risk groups are PTEN and TP53, respectively.

**Conclusions:**

Our results suggest that LNM plays an important role in the prognosis, and HMGB3 was potential as a biomarker for EC patients.

## Graphical abstract

Our results suggest that LNM plays an important role in the prognosis, and HMGB3 was potential as a biomarker for EC patients. By detecting the mutation of the risk signature, clinicians can accurately treat patients with targeted therapy, thereby improving their survival rate.

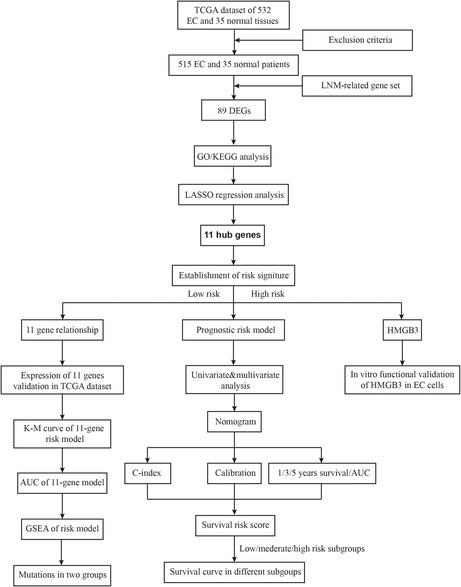

## 1 INTRODUCTION

Endometrial cancer (EC) is one of the most common gynecologic malignancies and also the sixth most common type of cancer in women worldwide. It was estimated that there were 382,069 new cases and 89,929 deaths in 2018.^1^ In China, EC ranks as the second most common cancer of the female genital system, and the five‐year overall survival rate is 55.1%.^2^ To stratify patients into distinct prognostic groups, EC and other cancers of the female gynecologic system are most commonly staged using guidelines provided by the International Federation of Gynecology and Obstetrics (FIGO) based on findings at both clinical examination and surgical exploration.^3^ However, traditional clinical criteria are not enough to predict EC prognosis accurately because the prognosis of patients with same clinicopathological factor varies differently. Therefore, it is imperative to emphasize the molecular changes that occur during endometrial cancer progression and develop novel predictive biomarkers to accurately estimate patient outcomes.

Machine learning is a form of artificial intelligence that can automatically analyze patterns from sample data, and make corresponding predictions. Due to its accuracy and predictive performance, the machine learning algorithm is used in different fields, including medical diagnostic and prognostic prediction.^4^ Recently, an in‐depth exploration of the public datasets, including TCGA and GEO, shed light on the heterogeneity in prognostic gene signatures of patients with similar clinicopathological features but distinct molecular features.^5^ With advancement in database mining, numerous biomarkers for EC have been identified as promising tools to classify tumors and predict cancer prognosis. For example, Wang et al. used the gene expression data associated with cellular glycolysis from the TCGA database to build a 9‐gene prognostic signature, and this signature identified patients with poor prognosis in EC.^6^ Zhou et al. integrated tumor mutation burden (TMB) with immune infiltrates to construct a TMB‐related signature (GFAP, EDN3, CXCR3, PLXNA4, SST), which had a better appraisal of prognostic and predictive factors and provided guidance of immunotherapy for EC.^7^

Lymph node metastasis (LNM) is a common problem in EC, which seriously affects the prognosis of patients and decreases the 5‐year survival. Therefore, it is increasingly urgent to understand mechanisms and find a specific therapeutic method for these patients. There are few reports concentrating on mRNA combination biomarkers for LNM of EC, so differentially expressed mRNA associated with LNM should be the key to the progression of EC.

To systematically investigate the roles of LNM in EC, we analyzed the expression profile of LNM‐related genes (), as well as normal tissues in The Cancer Genome Atlas (TCGA) database. Furthermore, we established and validated a multiple‐LRG‐combined expression signature for the prediction of EC patient outcomes. Multivariate Cox regression analysis suggested that risk score might be an independent prognostic indicator for the OS of EC patients and a prognostic nomogram model was established that could increase the accuracy of OS prediction. Our results also provide important insights into HMGB3 as a promising biomarker for the progression and provide novel perspectives for the therapeutic strategy in EC.

## 2 MATERIALS AND METHODS

### 2.1 Download of gene expression and clinical information

For endometrial cancers, information of gene expression was downloaded from the level‐3 gene‐expression information (FPKM normalized) of the TCGA‐UCSC cohort (https://portal.gdc.cancer.gov/), containing 35 normal samples and 532 tumor samples from patients with endometrial cancer. We then screened the corresponding clinical data and transcriptome data, and excluded the incomplete data. The collected clinicopathological data included age, menopause status, histology, lymph node metastasis, cancer status, peritoneal cytology, recurrence, stage, grade, survival status, and survival duration in days. Our research excluded any samples that had missing or insufficient data on age, menopause status, histology, lymph node metastasis, cancer status, peritoneal cytology, recurrence, stage, grade, survival status, and survival duration. Our study was in accordance with the publication guidelines provided by TCGA. LNM‐related genes (LRGs) gene set was downloaded from the Molecular Signatures Database (MSigDB) (http://www.gsea‐msigdb.org/gsea/msigdb/index.jsp).

### 2.2 Identification of DE‐LRGs and enrichment analysis

Differentially expressed transcriptome RNA‐sequencing of LRGs (DE‐LRGs) in 35 normal samples and 532 tumor samples were screened through “edgeR” package of R (version 3.6.1), and the screening standards were based on false discovery rate (FDR) < 0.05 and log2 |fold change| (log2FC) > 1. “Clusterprofiler” R package was used to analyze Gene Ontology (GO) functional enrichment and Kyoto Encyclopedia of Genes and Genomes (KEGG) pathway enrichment of DE‐LRGs. The results of GO annotation and KEGG pathway analyses were visualized by the “GOplot” package in R platform.

### 2.3 Establishment and validation of the prognostic model based on the DE‐LRGs

The least absolute shrinkage and selection operator (LASSO) regression analysis was used to establish the LRGs signature model. This model was used for subsequent evaluation and analysis of risk measures for the patients' risk values. We categorized these patients into high‐ and low‐risk groups according to the risk values. The risk score for each patient was computed using the following formula:

$$\text{Risk score}=\sum n_{i}=\sum \left(\text{Coefi}*x_{i}\right)$$

Coefi represented the coefficient and x_i_ represented the expression level of LRGs. Subsequently, the validity of the LRGs signature model was evaluated by analyzing the difference between high‐ and low‐risk groups. In the two subgroups, each patient's clinicopathological features and gene expression profiles were shown via the “pheatmap” and “survival” R packages. In addition, the Kaplan–Meier curve analysis and receiver operating characteristic (ROC) curves were performed to estimate the sensitivity and specificity of the prognostic signature.

The potential of the predictive model was validated in the testing cohort, in which RNAseq expression and clinical data were available for 30 surgically treated patients in our hospital. All samples were from patients between January 2018 and December 2020. Total RNA isolation and reverse transcription‐quantitative PCR procedures were performed as previously described.^8^ This research was approved by the Institutional Ethics Committee (Human Research) of our hospital and informed consent was obtained from the patients.

### 2.4 Gene set enrichment analysis (GSEA) analysis

GSEA (http://www.broad institute.org/gsea/index.jsp) was conducted to investigate the biological pathways of our prognostic gene signature.^9^ The collection of hallmarker.all.v6.1.symbols.gmt gene sets in Molecular Signatures Database (MSigDB, http://software.broadinstitute.org/gsea/msigdb/index.jsp) was taken as the reference gene sets in GSEA software. The standardized *p*‐value <0.05 and FDR <0.25 were considered to be significantly enriched when the samples were divided into high‐ and low‐risk groups.

### 2.5 Construction and evaluation the of the nomogram

Univariate and multivariate Cox regression analyses were used to determine whether the LRGs signature was independent risk factors for overall survival. Then we established a clinical practical nomogram to predict individual survival probability by the “rms” package of R. To evaluate whether the actual and the predicted survival in the nomogram are close to each other, calibration curves for predicting 3‐, 5‐, and 7‐year survival rate were drawn. The 45° line represented the best prediction.^10^ The accuracy of the nomogram was then performed with Kaplan–Meier survival analysis and the area under the tdROC curve (AUC). Harrel's concordance index (C‐index) was measured to validate the predictive ability of the nomogram.^11^

### 2.6 Experimental validation of HMGB3

Gene Expression Profiling Interactive Analysis (GEPIA) database (http://gepia.cancer‐pku.cn/) is an interactive website application that recruits the transcriptome data in TCGA and GTEx projects by integrating them in a widely accepted process.^12^ We inputted the hub gene into the GEPIA and validated these hub genes. Endometrial cancer cell line Ishikawa was obtained from ATCC (American Type Culture Collection). Ishikawa is originating from well‐differentiated endometrioid endometrial carcinoma, which is the most common type of EC. Therefore, we chose it to investigate the function of endometrial cancer cells. The cell line was cultured in DMED/F12 medium with 10% FBS according to the provider's instructions and incubated at 37°C with 5% CO_2_. HMGB3 siRNA plasmid was designed and synthesized by GenePharma (Shanghai, China). The plasmid was then transfected into the Ishikawa cell line. Opti‐MEM medium and Lipofectamine RNAiMAX reagent were used for the transfection of siRNA according to the manufacturer's instructions. Then total protein of EC cell line in different groups were extracted and used for western blot as described in this article.^13^ The knockdown efficiency was proved by western blot using primary antibodies against HMGB3 (1:500; Cat. No. AF5507; R&D Systems, Inc.), and GAPDH (1,1,000; Cat. No. 5174S; Cell Signaling Technology). Cell proliferation assays were performed by Cell Counting Kit‐8 (Dojindo Molecular Technology). 10 μl CCK8 solution was added into each 96‐well plate and incubated for 2 h. The optical density was measured at 450 nm by microplate spectrophotometer.

By evaluating the effect of HMGB3 on the metastatic and invasive ability of EC cells, we conducted a gap closure and transwell invasion assay. Cells were seeded in the 6‐well plate and cultured to about 85% density confluence. A straight wound was scratched carefully using 200 μl sterile pipette tips. After scratching, cell debris was washed away with PBS. For transwell assay, at a density of 1 × 10^5^ per well, cells were seeded into the upper chamber, and then incubated for 24 h. Cancer cells were fixed with 4% paraformaldehyde for 30 min, stained with 0.1% crystal violet for 5 min, washed 3 times with PBS, and counted in 6 fields under the microscope. Images were taken at 0 h and 24 h, and analyzed by Image J software (Rawak Software, Inc.). All of the experiments were repeated 3 times.

### 2.7 Immunohistochemistry (IHC)

Five resected normal endometrial tissues and five EC tissues were obtained for immunohistochemistry. The diagnosis was confirmed pathologically. The immunohistological (IHC) analysis for HMGB3 in EC and normal endometrial tissue was performed as previously described.^14^

### 2.8 Mutation analysis

The mutation data of EC patients were obtained from the TCGA database mentioned above. To compare the mutational loading between low‐ and high‐risk groups, we extracted the mutation data of somatic variants from Mutation Annotation Format (MAF), and analyzed it by the MAF tools package.^15^

### 2.9 Statistical analysis

Data are expressed as means ± SD. For data analyses, two‐tailed Student's *t*‐tests and Wilcoxon matched‐pairs tests were performed using R software or SPSS 25.0. In all tests, values of *p* < 0.05 were considered statistically significant.

## 3 RESULTS

### 3.1 Differentially expressed LRGs in EC

**FIGURE 1:**
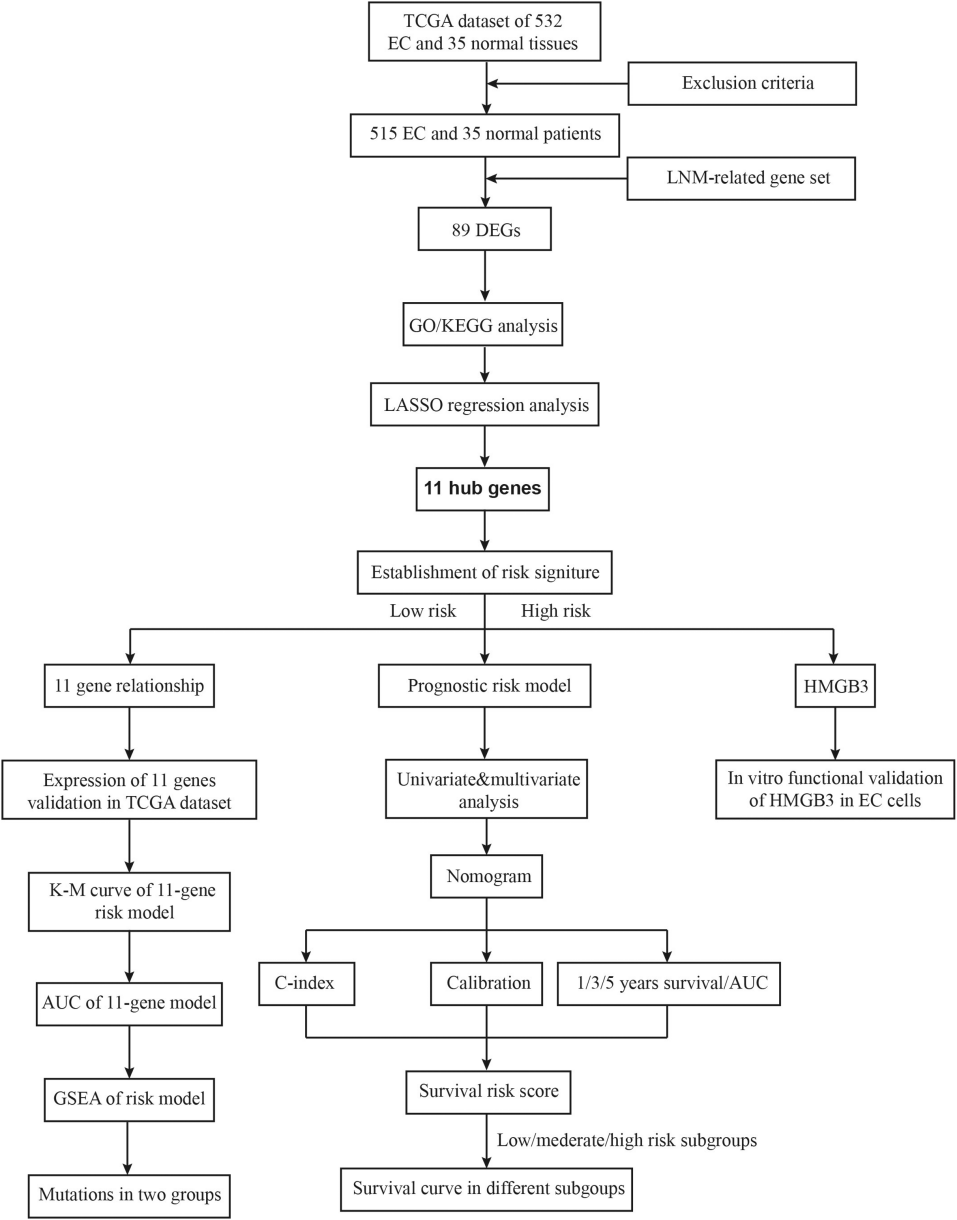
Flowchart for identifying the LNM‐related prognostic signature.

**FIGURE 2:**
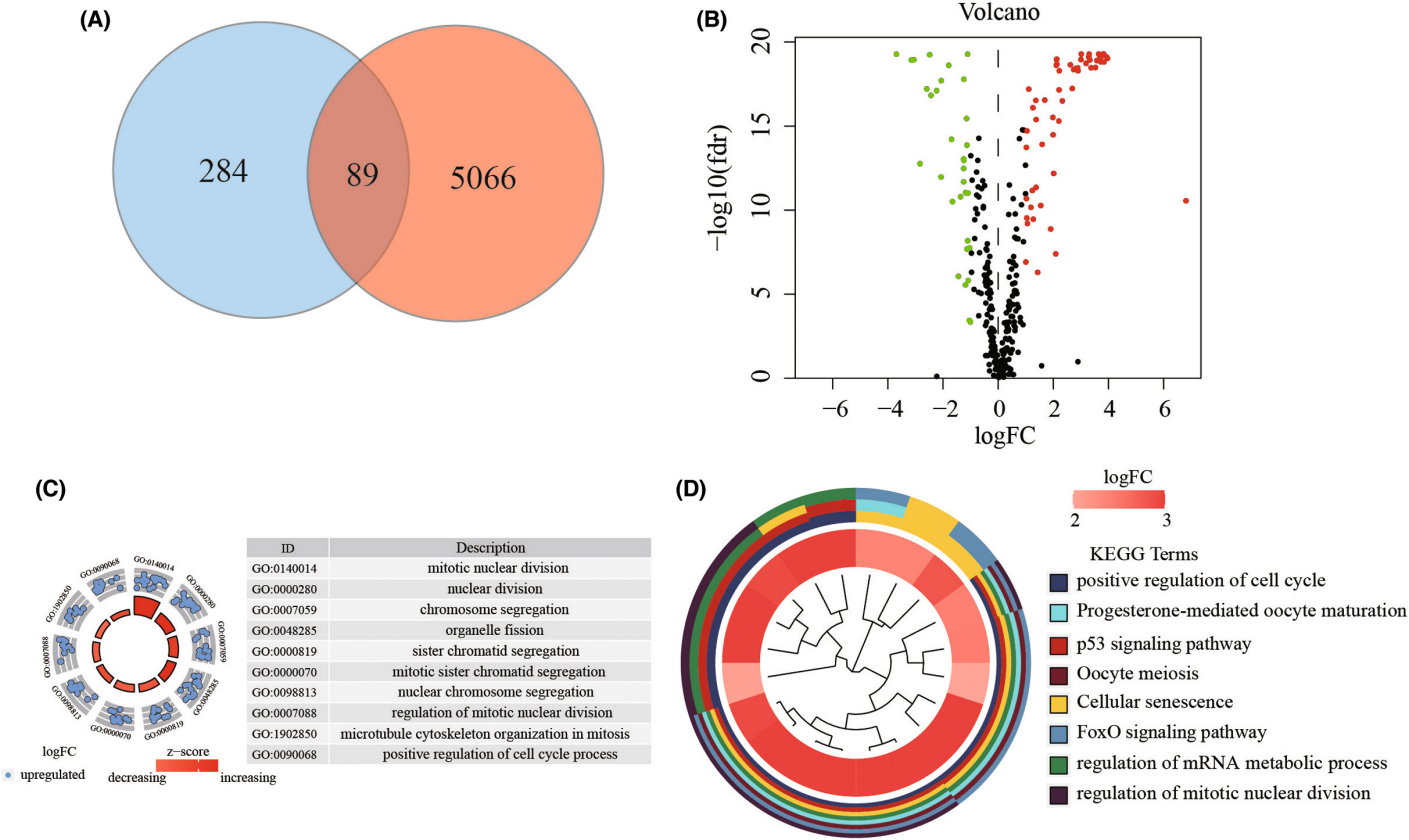
Identification and functional analysis of LRGs in EC. (A). The intersect LNM‐related genes were associated with DEGs between normal and EC tissues. (B). Volcano plot was drawn to show the differentially expressed LNM‐related genes. (C). Enrichment analysis reveals the top 10 GO terms and (D). KEGG pathways.

The process for this study is shown in Figure 1. A total of 515 patients were involved in the development and validation of the prognostic signature, including 35 normal tissues. First, we performed the Wilcoxon test with a log_2_FC >1 and *p* < 0.05 to detect the differentially expressed genes (DEGs). Then, 5155 DEGs were found between 35 normal samples and 515 tumor samples. Next, we downloaded the list of LRGs from MSigDB. These LRGs intersected with the DEGs, and 89 differentially expressed LRGs were obtained (Figure 2A), including 32 down‐regulated and 57 up‐regulated genes (Figures 2B). The DE‐LRGs list, including log_2_FC and the adjusted *P*‐values of each gene was provided in Table S1. Afterwards, we performed GO and KEGG pathway analysis for the DE‐LRGs and the top 10 GO and KEGG pathway enrichment terms shown in Figures 2C‐D.

GO analysis revealed that the DE‐LRGs were mainly enriched in mitotic nuclear division, nuclear division, chromosome segregation, and so on. The KEGG analysis indicated that the genes were mainly involved in positive regulation of the cell cycle, progesterone‐mediated oocyte maturation, p53 signaling pathway, et al. Thus, through the combined analysis, we revealed 89 DE‐LRGs that were significantly associated with EC. Most of the DEGs were associated with the cell cycle and p53 pathway.

### 3.2 Identification of prognostic LRGs‐related DEGs

**FIGURE 3:**
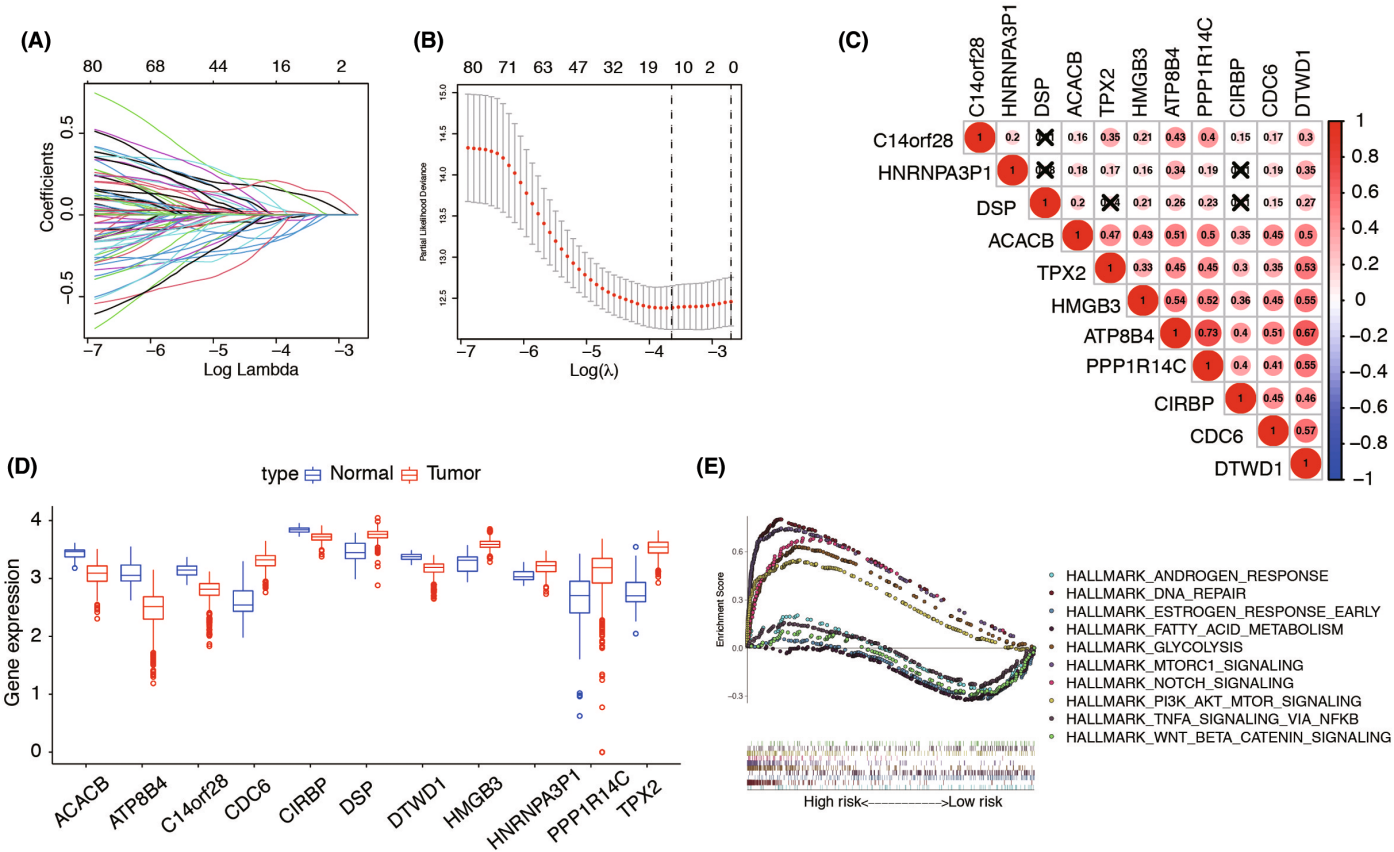
Construction of risk signature with LRGs. (A). LASSO coefficients. (B). Plots of the 10‐time cross‐validation for tuning parameter selection in the least absolute shrinkage and selection operator (LASSO) model. The dashes signify the value of the minimal error and greater λ value. (C). Spearman correlation analysis of the 11 LNM‐related genes. (D). The expression of 11 LRGs between TCGA endometrial cancer (EC) and normal tissues. (E). GSEA showed significant enrichment of the tumor‐related signaling pathways.

To further screened out the LRGs with potential prognostic value for EC patients, the LASSO regression model was used to select key prognosis‐associated genes. In the LASSO‐penalized Cox regression, as log λ (a tuning parameter) changed, the corresponding coefficients of certain genes were reduced to zero, suggesting that their effects on the model could be omitted because they were shrinking parameters (Figure 3A). Following cross‐validation, 11 genes (C14orf28, HNRNPA3P1, DSP, ACACB, TPX2, HMGB3, ATP8B4, PPP1R14C, CIRBP, CDC6, DTWD1) achieved the minimum partial likelihood deviance and were identified as key prognostic LRGs‐related genes in OS model (Figure 3B). The corresponding coefficients of the 11 genes are shown in Table 1. As described in the methods. The calculation formula = (ACACB × 0.1335) − (ATP8B4 × 0.0144) − (C14orf28 × 0.0007) − (CDC6 × 0.0727) − (CIRBP × 0.1112) + (DSP × 0.0196) − (DTWD1 × 0.0318) + HMGB3 × 0.1201) − (HNRNPA3P1 × 0.1167) + (PPP1R14C × 0.0185) + (TPX2 × 0.1833). The correlations between these genes were calculated in EC using the Spearman correlation analysis. We found they were significantly relevant. For instance, expression levels of DTWD1 and ABACB, ATP8B4 and CDC6, ATP8B4 and HMGB3 genes were closely correlated with each other (Figure 3C). The expression of these 11 genes in tumor and adjacent normal tissues were shown in Figure 3D. The results showed that the expression of ACACB, ATP8B4, C14orf28, CIRBP, and DTWD1 were higher in normal tissues, and the content of CDC6, DSP, HMGB3, HNRNPA3P1, PPP1R14C, and TPX2 were higher in tumor tissues. Furthermore, the transcript message of patients stratified by risk score into high‐ and low‐ risk subgroups were analyzed by GSEA. In the prognostic model, representative hallmark in high‐risk patients were “androgen response”, “DNA repair”, “estrogen response early”, “fatty acid metabolism”, “glycolysis”. What's more, “mTORC1 signaling”, “Notch signaling”, “PI3K‐AKT–mTOR signaling”, “TNFA signaling via NFKB”, “WNT‐β‐catenin‐ signaling” were enriched in low‐risk group patients (Figure 3E).

**TABLE 1:** Eleven lymph node metastasis associated genes and corresponding coefficient value

LNM associated genes	Coefficient
ACACB	0.13351004
ATP8B4	−0.0144339
C14orf28	−0.0007171
CDC6	−0.0727459
CIRBP	−0.1112859
DSP	0.01966864
DTWD1	−0.0318811
HMGB3	0.12010494
HNRNPA3P1	−0.1167104
PPP1R14C	0.01855752
TPX2	0.18337651
Risk score	Low: <3.75
	High: ≥3.75

### 3.3 Validation and the efficacy of the 11‐LRGs prognostic signature

**FIGURE 4:**
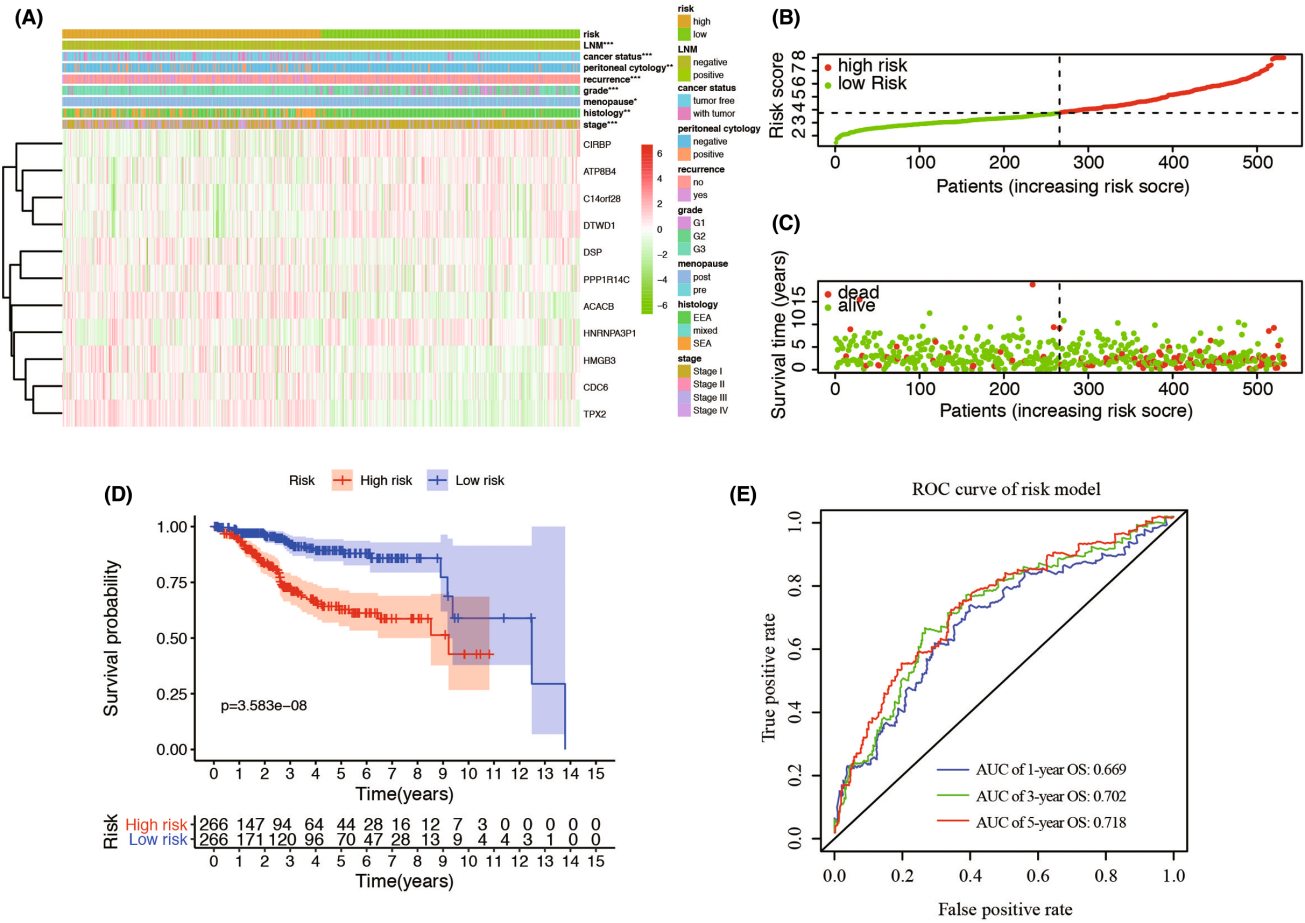
Prognostic analysis of the risk model in the TCGA patients. (A). The heatmap shows the expression of the 11 genes in high‐risk and low‐risk group of EC. The distribution of clinicopathological characteristics was compared between the high‐risk and low‐risk groups. (B–C). The distributions of the five‐gene signature and survival status of the patients in the risk signature. (D). Kaplan–Meier survival analysis with patients in low‐ and high‐risk groups. (E). 1‐, 3‐, 5‐year survival time‐dependent receiver operating characteristic (ROC) curve. *p* < 0.05; **, *p* < 0.01; ***, *p* < 0.001. ROC, receiver operating characteristic; AUC, area under the ROC curve. E. The 1‐, 3‐, and 5‐year AUC of ROC curves.

Based on the mean risk score from risk signature, the patients were divided into high‐risk and low‐risk groups. Then, the expression of the 11 genes in low‐ and high‐risk patients in the TCGA dataset was also demonstrated in the heatmap (Figure 4A). We found significant differences between the high‐ and low‐risk groups associated with LNM, cancer status, peritoneal cytology, recurrence, grade, menopausal status, stage, and stage (all *p* < 0.05). Then risk score of each individual and survival status were ranked and displayed on the dot plot, which showed significant differences in OS between the groups (Figure 4B–C). Likewise, the Kaplan–Meier curve analysis demonstrated that the OS of the high‐risk group was significantly shorter than that of the low‐risk group (*p* = 3.583e‐08) (Figure 4D). ROC curve analysis revealed that the area under the ROC curve (AUC) of 1‐, 3‐, 5‐year survival of the prognostic LRG model was 0.669, 0.702, and 0.718 (Figure 4E). Furthermore, we analyzed the 11 genes for different statuses of LNM, respectively. We found that expression levels of C14orf28, CIRBP, DTWD1, HMGB3, and TPX2 were significantly different (Figure S1). The external validation of risk model showed similar results among the whole cohort. The expression pattern in our center was the same as that in TCGA risk model (Figure S2A–C). Overall survival and recurrence‐free survival curves showed that patients in high‐risk score had a worse outcome in both groups (Figure S3). The performance of heatmap for the 30 patients also had a similar result as that in TCGA patients (Figure S4). Above all, these results revealed that the LRGs risk model could be served as an effective and accurate prognostic signature in EC.

### 3.4 Nomogram building and validation

**FIGURE 5:**
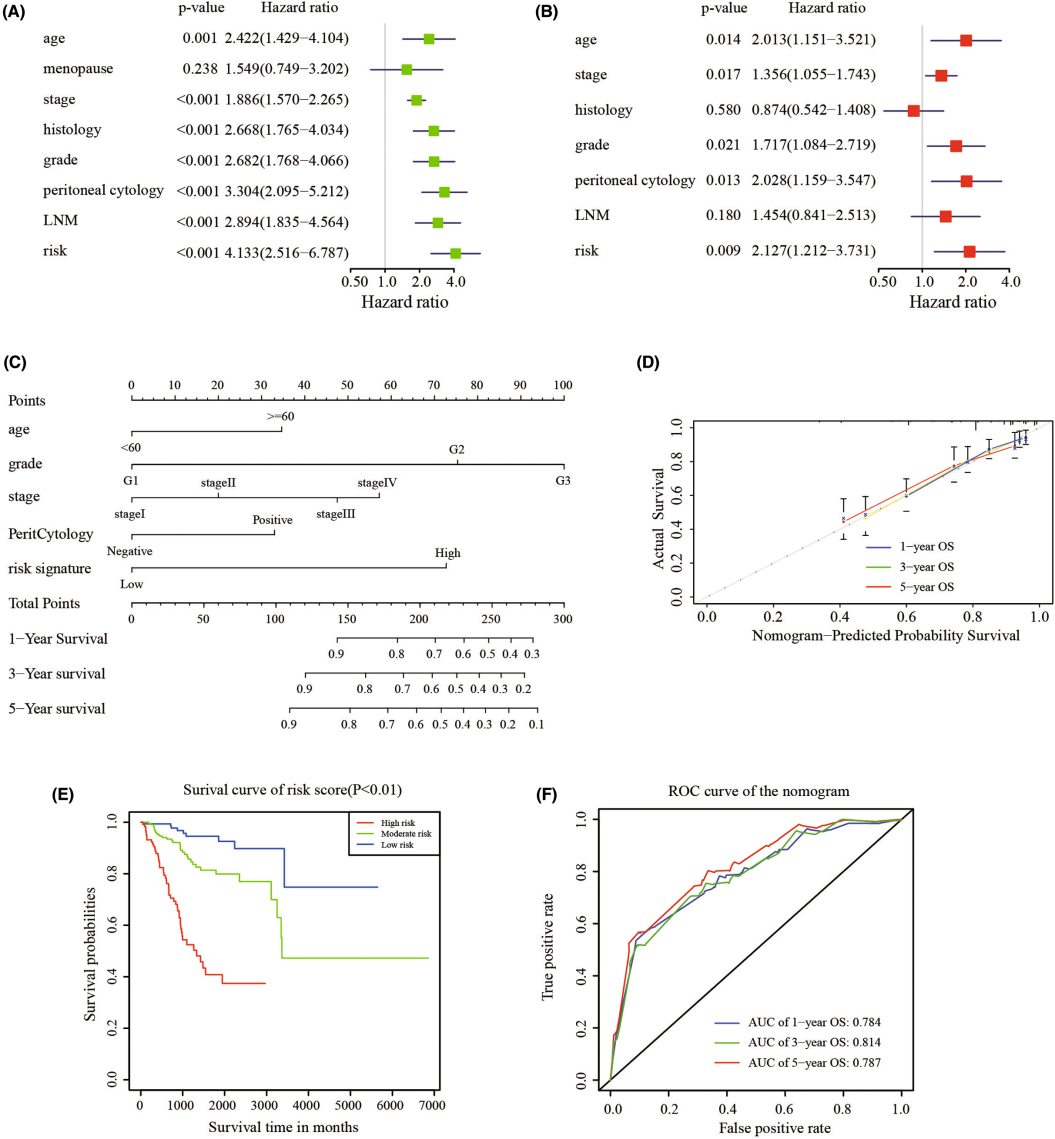
Construction and validation of nomogram. (A‐B). Univariate and multivariate Cox analyses of cervical cancer. (C). Nomogram used to predict prognosis in patients with endometrial cancer at 1, 3, and 5 years. (D). Calibration curves for the nomogram at 1‐. 3‐, and 5‐year overall survival. (E). Survival curve of patients in low‐, moderate‐, and high‐score according to the total score of the nomogram. (F). ROC curve of 1‐, 3‐, 5‐year survival depend on the nomogram.

For supplying the clinicians with a practical formula to estimate EC patients' survival probability, a comprehensive prognostic nomogram based on the patients' risk scores and clinical features was built. First of all, univariate and multivariate Cox regression analyses of survival were performed by the risk signature and related clinicopathological factors (age, menopausal status, stage, histology, grade, peritoneal cytology, LNM, and 11‐gene risk model) in the TCGA dataset were used to determine whether the 11‐gene risk score can be used as an independent prognostic factor. Both univariate and multivariate analyses showed that the risk model could be used as a prognostic indicator (*p* < 0.001, Figure 5A–B). Afterwards, five independent prognostic parameters, including age, grade, stage, peritoneal cytology, and 11‐gene risk model were integrated into the nomogram (Figure 5C). The specific score of each factor was shown in Table 2. The C‐index of the model was 0.769 (C‐index = 0.712 for clinical prognostic factors alone). The calibration plots showed excellent consistency between the nomogram predictions and actual observations in terms of the 1‐, 3‐, and 5‐year survival rates in the TCGA cohort (Figure 5D). Furthermore, we divided the cohort into 3 subgroups (low‐score, moderate‐score, and high‐score groups) evenly according to their risk scores from the nomogram. The survival curve of the high‐score group had a worse OS than the moderate‐ and low‐score groups (Figure 5E). In order to find out whether the risk signature was an effective prognostic indicator, tdROC was plotted. Similar to the performance in the cohort, the AUCs were 0.784, 0.814, and 0.787 for 1‐, 3‐, and 5‐year survival time, respectively (Figure 5F). These results found that the 11‐gene LRGs model improved the predictive accuracy of OS in EC patients.

**TABLE 2:** Corresponding risk score for each variable and total score

Variables	Category	Score
Age	<60	0
	≥60	35
Grade	G1	0
	G2	75
	G3	100
Stage	Stage I	0
	Stage II	20
	Stage III	45
	Stage IV	55
Peritoneal cytology	Negative	0
	Positive	32.5
Risk signature	Low	0
	High	72.5
Total score	Low risk	0–110
	Moderate risk	112.5–207.5
	High risk	≥212.5

### 3.5 Clinical experimental validation

**FIGURE 6:**
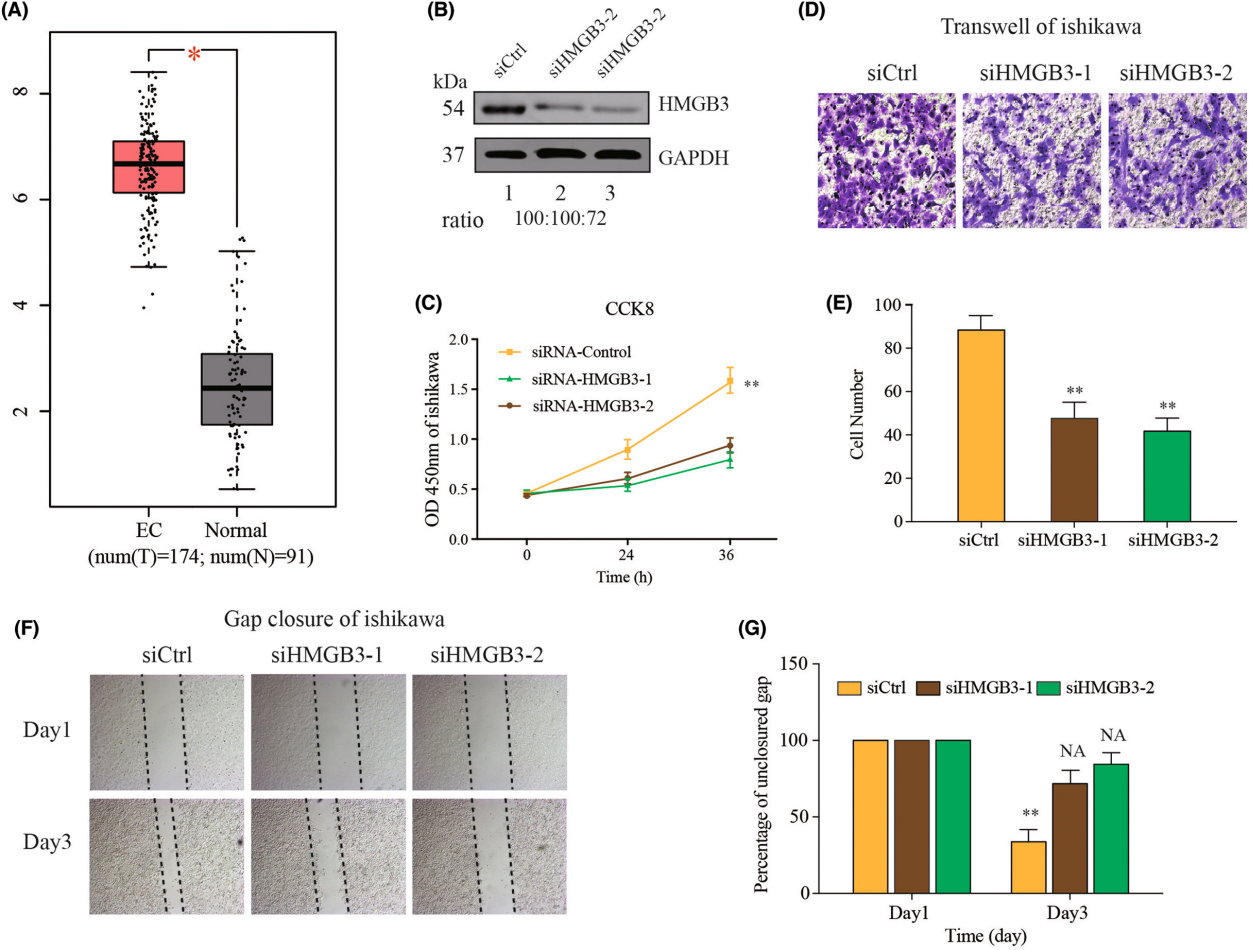
In vitro functional validation of the HMGB3. (A). The relative expression of HMGB3 in GEPIA. (B). Knockdown efficiency of HMGB3 by two small interferon RNA transfection. (C). Proliferative effect of HMGB3 on Ishikawa evaluated by Cell Counting Kit‐8 tests. (D). Effects of HMGB3 on the invasion of Ishikawa cells evaluated by Transwell assays. (E). Statistical analysis of the Transwell invasion. (F) Effects of MMP12 on the migration of Ishikawa cells evaluated by gap closure assays. (G) Statistical analysis of the gap closure. *p < 0.05, **p < 0.01.

First of all, the differential expression of 11 genes was validated based on GEPIA website (Figure S5), including HMGB3 (Figure 6A). We knocked down the expression of HMGB3 in Ishikawa cell line and conducted the following functional experiments. Then western blot was performed to examine the efficiency of knockdown. The results suggested that the expression of MMP12 in the siRNA group was significantly down‐regulated compared with the negative control group (Ctrl) and the blank control group (siCtrl) in Ishikawa cells (*p* < 0.01, Figure 6B). CCK‐8 assay suggested that the proliferation rate of Ishikawa was significantly decreased in HMGB3 knockdown group (Figure 6C). Transwell and gap closure assays were performed to investigate the effects of HMGB3 on the invasion and metastatic behaviors of EC cells in vitro. The results demonstrated that Ishikawa in HMGB3 knockdown group exhibited significant declines in invasion capabilities (Figure 6D–E) and migration (Figure 6F–G) compared with respective control groups. Immunohistochemistry () detection of HMGB3 was performed on five EC and five normal endometrial tissues. In Figure S6, the colors yellow and brown indicated positive expression of the marker. HMGB3 was found to be highly expressed in EC tissues.

These results suggested that HMGB3 played a crucial role in the progression of EC cells.

### 3.6 The immune‐related risk signature and mutation profile

**FIGURE 7:**
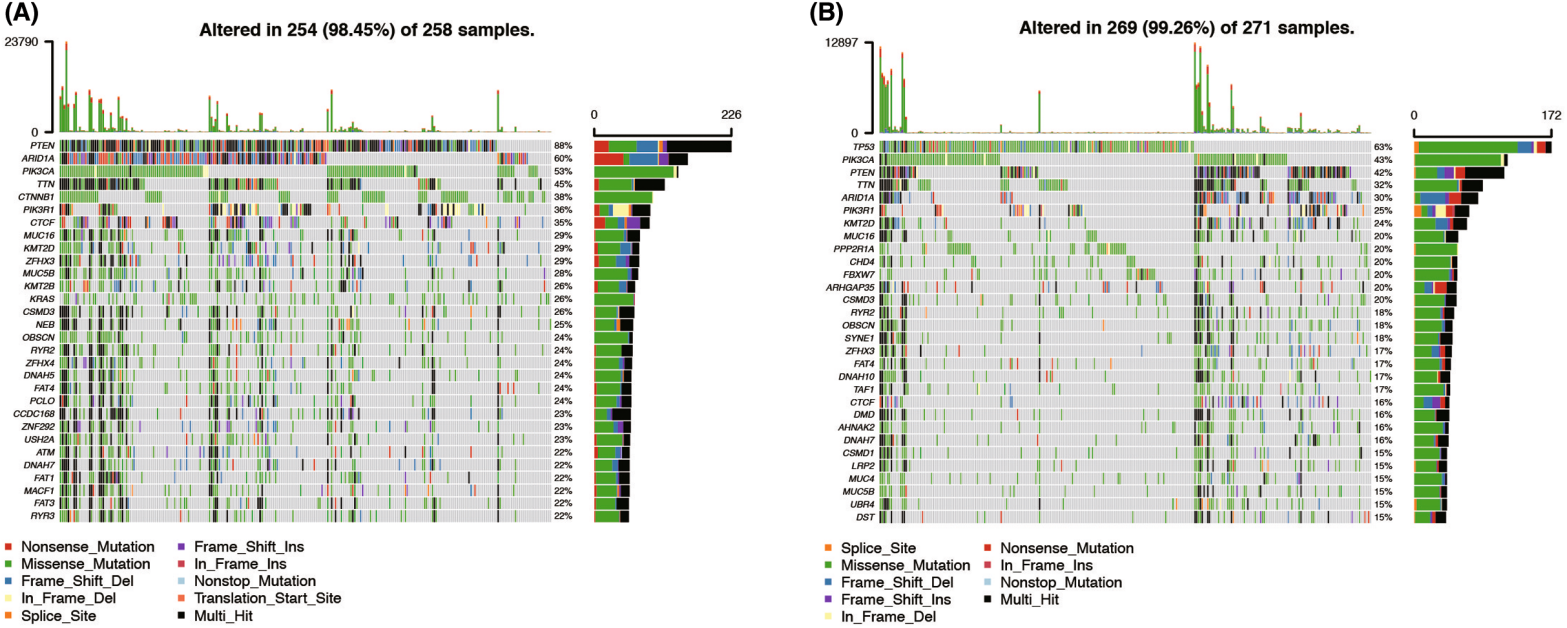
Landscape of mutation genes in low‐ and high‐risk groups. Waterfall plot showing mutation profiles of each gene in each endometrial cancer sample. (A). Low risk group. (B). High risk group.

Gene mutations are an important cause of tumorigenesis and development. Hence, we evaluated tumor mutation burden (TMB) of patients in low‐ and high‐risk groups with somatic mutation data. In the risk model, the low‐risk group had somatic mutations in the following order: PTEN> ARID1A > PIK3CA > TTN > CTNNB1 > PIK3R1 > CTCF> MUC16 > KMT2D > ZFHX3 (Figure 7A). Meanwhile, in the high‐risk group, somatic mutations were listed in the following order: TP53 > PIK3CA > PTEN> TTN > ARID1A > PIK3R1 > KMT2D > MUC16 > PPP2R1A > CHD4 (Figure 7B). The TMB scores of patients stratified by the LRGs prognostic model were therefore investigated. The *t* test demonstrated a significant difference between the low‐risk and high‐risk groups (*p* < 0.01, Figure S7). These results indicated that different risk groups in the LRGs risk model had different TMB features.

## 4 DISCUSSION

The prognosis of EC is great, however, extensive heterogeneity has been reported in a number of studies associated with recurrent disease and high mortality.^16^ Recent studies showed that clinicopathological features such as age and metastatic diagnosis are not sufficient to precisely predict the outcome of patients with cancer. Thus, there remains a need to elucidate the molecular mechanisms underlying tumor progression or prognosis. Accumulating evidence shows that a comprehensive understanding of EC requires attention not only to clinicopathological features but also to the gene expression. An increasing number of evidence showed that EC patients with lymph node metastasis always exhibited poor responses to standard treatments and thus tend to have poor clinical outcomes.^17^ For patients with EC, the method of sentinel lymph node (LN) biopsy has been developed during these years. The overall detection rate of sentinel LN was 95%, with 74% positive bilaterally.^18^ In addition, the identification of risk factors is also an important measurement for conducting lymphadenectomy.

In this study, we performed a comprehensive analysis of lymph node metastasis‐related genes (LRGs) in EC and linked the data to clinical outcomes and prognosis of patients with EC. First, we systematically studied the LRGs in EC and identified 89 differentially expressed LRGs (DE‐LRGs). Based on multivariate Cox coefficients derived from LASSO analysis, we developed a 11‐gene signature‐based risk score model associated with overall survival in EC patients. Subsequently, both time‐dependent ROC curves and Kaplan–Meier plots revealed that the risk signature outperformed in the prediction of OS for EC patients. Validation in our own patients also indicated that this risk signature was stable and persuasive. By combining the established clinicopathological factors with the signature, we developed a nomogram to predict the survival probability of EC patients. The predictive power was measured by the time‐dependent area under the ROC curve (AUC), and the result showed that the integrated nomogram model had higher predictive power than other models. Lastly, HMGB3 was identified as an oncogene to promote the proliferation and migration of EC cells.

Finally, we analyzed and compared the somatic mutation of low‐ and high‐risk group patients to visualize the mutation annotation format of the two groups. PTEN was the highest mutated gene in low‐risk group, and TP53 was the highest in high‐risk group. Longstanding molecular observations implicate PTEN inactivation as a major driver of endometrioid carcinomas; TP53 inactivation as a major driver of most serous carcinomas, some high‐grade endometrioid carcinomas, and many uterine carcinosarcomas; and inactivation of either gene as drivers of some clear cell carcinomas. Dysfunction of p53 in endometrial cancer is closely associated with TP53 mutation. TP53 mutation is detected in about 25% of all endometrial cancer patients.^19^ One study developed OB‐P702,^20^ a telomerase‐specific replication‐competent adenovirus with the expression of wild‐type p53. Telomerase is activated in many malignant tumors, including gynecologic carcinomas.^21^ OBP‐702 can replicate in cells with telomerase activity. OPB‐702 induced p21 suppression by E1A‐mediated miR‐93/106b upregulation, leading to p53‐mediated apoptosis and autophagy.^22^

As far as we know, this is the first study concentrating on lymph node metastasis‐related genes and prognostic risk signature construction in EC. In this study, we investigated a set of genes associated with lymph node metastasis and then constructed a prognostic signature predicting the overall survival of this set of genes. There were other studies concentrating on the lymph node metastasis and EC. However, some studies tried to construct models to predict lymph node metastasis in early EC.^23^, ^24^, ^25^Another study constructed a 15‐miRNA signature to predict LNM in EC patients, and hub miRNAs in signature contributed to EC progression via mitotic cell cycle.^26^

However, we might carry out the differential analysis between patients with good and poor outcomes, and then further screen metastasis‐related genes from the differentially expressed genes.^26^ There are studies using the LNM‐related genes to establish the prognostic model in other cancers. Huang et al. found 16 DEGs by analyzing six patients with and 18 patients without LNM for RNA sequencing in their own center and validated the predictive model by TCGA. Receiver operating characteristic curve analysis revealed that this model can predict LNM. The accuracy, negative, and positive predictive values were 84.7%, 98.1, and 44.4%, respectively. However, they did not explored the function and potential therapeutic targets and pathways in the following analysis.^25^ In our study, we not only evaluated the predictive value of risk model, but also validated the function of hub gene. One study proved a four‐gene signature can be used as a combined biomarker for independent prognosis of colorectal cancer. In this model, high‐risk group patients were associated with neuroactive ligand‐receptor interaction and estrogen signaling pathway. AUC of the overall survival of the risk model reached 0.730.^27^ Wang et al. developed a robust mRNA signature as an independent factor to effectively classify LUAD patients with LNM into low‐ and high‐risk groups using TCGA database, and validated by an external cohort from Gene Expression Omnibus (GEO). GSEA showed the hallmarkers correlated with the high‐risk group were EMT, hypoxia, and MYC targets.^26^ The latest study recognized CDKN2B‐AS1 as a molecular target associated with immune infiltration and prognosis and provide new insights into the development of molecular therapies and treatment strategies against EC. AUC of the risk model was 0.687, which is not as good as ours (AUC = 0.718 for 5‐year survival).^28^ These studies including ours all suggested that LNM‐related genes were promising for many types of cancer in the prediction of overall survival.

To study the potential molecular mechanism of prognostic effects of the 11‐gene signature, GSEA analysis was conducted. The results of GSEA showed that the signature‐identified high‐risk group was significantly correlated with certain hallmarks of cancer, such as fatty acid metabolism, glycolysis, Notch signaling pathway, and PI3K‐AKT–MTOR signaling pathway, indicating the potential molecular mechanisms underlying the lethal tendency of patients in the risk signature. Metabolic disorders had long been reported in EC, and over‐expression of fatty acid synthase is associated with cancer progression and upper body fat distribution in EC.^29^ Glycolysis of cellular respiration is a complex reaction and is the first step in most carbohydrate catabolism, which was proved to participate in EC. One study used nine glycolysis‐related genes to predict the survival of patients with EC, and the predictive accuracy of overall survival proved to be high for EC patients (AUC of 1‐year OS = 0.763).^30^ Evolutionary conserved Notch signaling pathway regulated diverse cellular processes including proliferation, differentiation, and cell invasion. Accumulating evidence links aberrant Notch signaling with EC. For example, FOXA1 promotes cell proliferation by androgen receptor and activates the Notch pathway in EC.^31^ Activation of the PI3K/AKT/mTOR pathway reversed FAM83B knockdown‐induced autophagy promotion and inhibition of proliferation, migration, and invasion in EC cells. This results indicated that proliferation and metastasis cells inhibited autophagy via activating the PI3K/AKT/mTOR pathway in EC cells.^32^

Molecular classification of EC has been shown to be reproducible and associated with clinical outcomes.^33^, ^34^ An integrated genomic‐pathologic classification of EC has been proposed by, which defined four major classes of EC as POLE‐ultramutated, microsatellite instability–hypermutated (MSI‐H), copy‐number‐low (CNL), and copy‐number‐high(CNH). It also needs to be noteworthy that some of these genes have been reported in previous studies of cancer.^19^ As for the characteristics of these signature genes, higher expression levels of ACACB, DSP, HMGB3, PPP2R14C, and TPX2 are associated with poor prognosis. On the other hand, higher expression levels of the remaining C14orf28, HNRNPA3P1, ATP8B4, CIRBP, CDC6, and DTWD1 are associated with longer OS.

ACACB was associated with HER2‐positive breast cancer with brain metastasis.^19^ TPX2 represents a novel prognostic factor for esophageal cancer, and he 5‐year overall survival rate of TPX2 high expression group was significantly lower than that of TPX2 low expression group.^35^ Several studies have demonstrated that TPX2 also played an important role in the development of prostate cancer,^36^ non‐small cell lung cancer,^37^ and breast cancer.^38^ Loss of CIRBP expression is correlated with the malignant progression and poor prognosis in nasopharyngeal carcinoma.^39^ Among the 11 coding genes, HMGB3 was widely reported to promote malignant progress and predict poor survival in various cancer types.^40^, ^41^, ^42^ In addition, Gu et al. reported that HMGB3 silence could inhibit the cell proliferation in vitro and suppress tumor growth in vivo levels. The antitumor effects of HMGB3 silence were mediated by interacting with the HIF1α.^43^ But the expression and function of HMGB3 in EC cells were still ambiguous. What's more, high mobility group protein (HMG) is considered the second most abundant cellular protein and plays a global role in the construction of chromatin domains.^44^ By interacting with nucleosomes, transcription factors, nucleosome remodeling complexes, and histone H1, HMG promotes transcriptional fine‐tuning in response to rapid environmental changes. In the progression of EC cells, different environmental changes are crucial for EC cells to adapt.^45^ Therefore, we further explored the molecular function of HMGB3 in EC cell line Ishikawa. The results revealed that silencing expression of HMGB3 inhibited proliferation, migration, and invasion of EC cell lines.

The relationship between the mutation profile and the signature was also performed to explore the possible mechanisms of the signature's prognostic value. Mutation profiles of the 11‐LRG signature high‐risk group and low‐risk group were different. The most frequently mutated genes in the low‐risk and high‐risk groups are PTEN and TP53, respectively. Phosphatase and Tensin homolog (PTEN) is a tumor suppressor gene. Loss of its function is the most frequent genetic alteration in endometrioid endometrial cancers (70–80%) and high‐grade tumors (90%). However, TP53 mutations are considered a surrogate biomarker of the serous‐like ‘copy number high’ molecular subtype of endometrial carcinoma (EC).^46^ Understanding the complex interaction of mutated genes in endometrial cancer will help to better select patients that are likely to respond to some of the new and costly targeted therapies.

In this study, we constructed a valid LNM‐related risk signature that can comprehensively predict the survival of EC patients. Furthermore, the proliferation and metastasis of HMGB3 were also validated in vitro, and HMGB3 is potential to become a new biomarker for EC patients. To the best of our knowledge, there are no studies exploring the function of HMGB3 in EC, and this is the first LNM‐related predictive model for EC patients using RNA‐sequencing technology. Additionally, our signature can predict patient survival and disease progression. Despite these promising results, there are several limitations in this study. First, the signature was constructed from public data and retrospective studies. Second, in this TCGA‐EC cohort, the proportion of Chinese patients was small. It is unclear whether this signature will function effectively for Asian patients. Future studies should incorporate a larger number of Chinese EC samples. Finally, external validation is also needed in other centers or datasets.

## 5 CONCLUSION

In conclusion, we identified differentially expressed LNM‐related genes that may involve in the prognosis in EC patients. These genes have significant values in predicting the patients' OS and HMGB3 may be a therapeutic target for EC. Further studies are necessary to verify these results in our study.

## CONFLICT OF INTEREST

The authors have no conflicts of interest to disclose.

## AUTHOR CONTRIBUTION

**Conceptualization:** Hong Wu, Liping Yang.; **Data curation:** Haiqin Feng; **Formal analysis:** Haiqin Feng, Xiaoli Miao; **Investigation:** Jiancai Ma, Cairu Liu; **Methodology:** Hong Wu, Lina Zhang; **Resources:** Liping Yang, Xiaoli Miao; **Validation:** Haiqin Feng; **Writing – original draft:** Hong Wu; **Writing – review & editing:** Liping Yang, Hong Wu.

## ETHICAL STATEMENT

The authors are accountable for all aspects of the work in ensuring that questions related to the accuracy or integrity of any part of the work are appropriately investigated and resolved.

## STATEMENT

The study conformed to the provisions of the Declaration of Helsinki (as revised in 2013), and we confirm that all figures and tables are original.

## Supporting information


Figure S1

Figure S2

Figure S3

Figure S4

Figure S5

Figure S6

Figure S7

Table S1

Table S2

## Data Availability

The database supporting the conclusions of this article is available in the TCGA database database.
