# Supplementary figures and images for: Construction and validation of a prognostic model based on 11 lymph node metastasis‐related genes for overall survival in endometrial cancer
Source: Cancer Med. 2022 Jul 2;11(23):4641–55. doi: 10.1002/cam4.4844 (PMC9741985; doi:10.1002/cam4.4844)

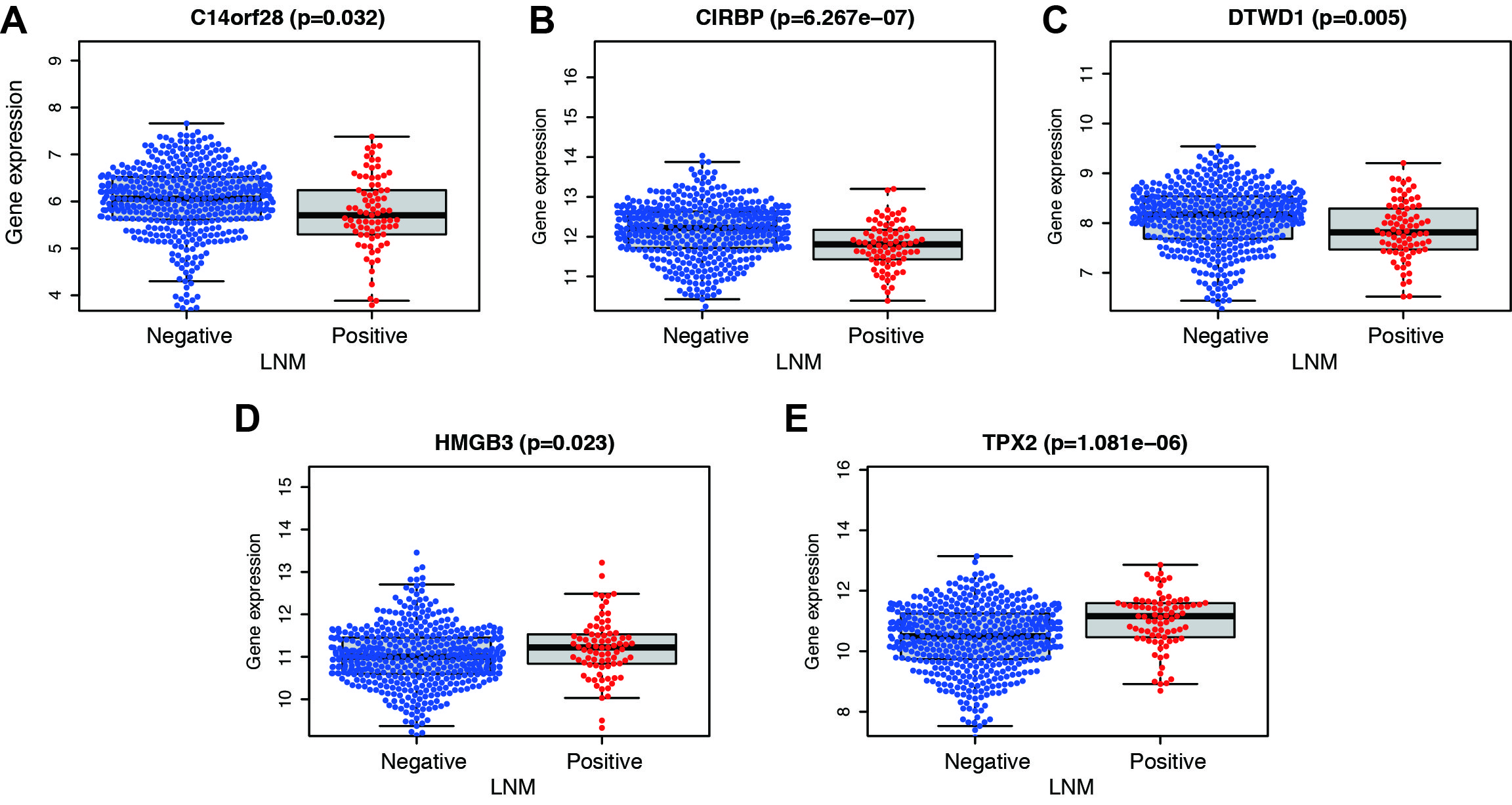

Supplement: Supplementary file 1 — Figure S1 [file CAM4-11-4641-s004.jpg]

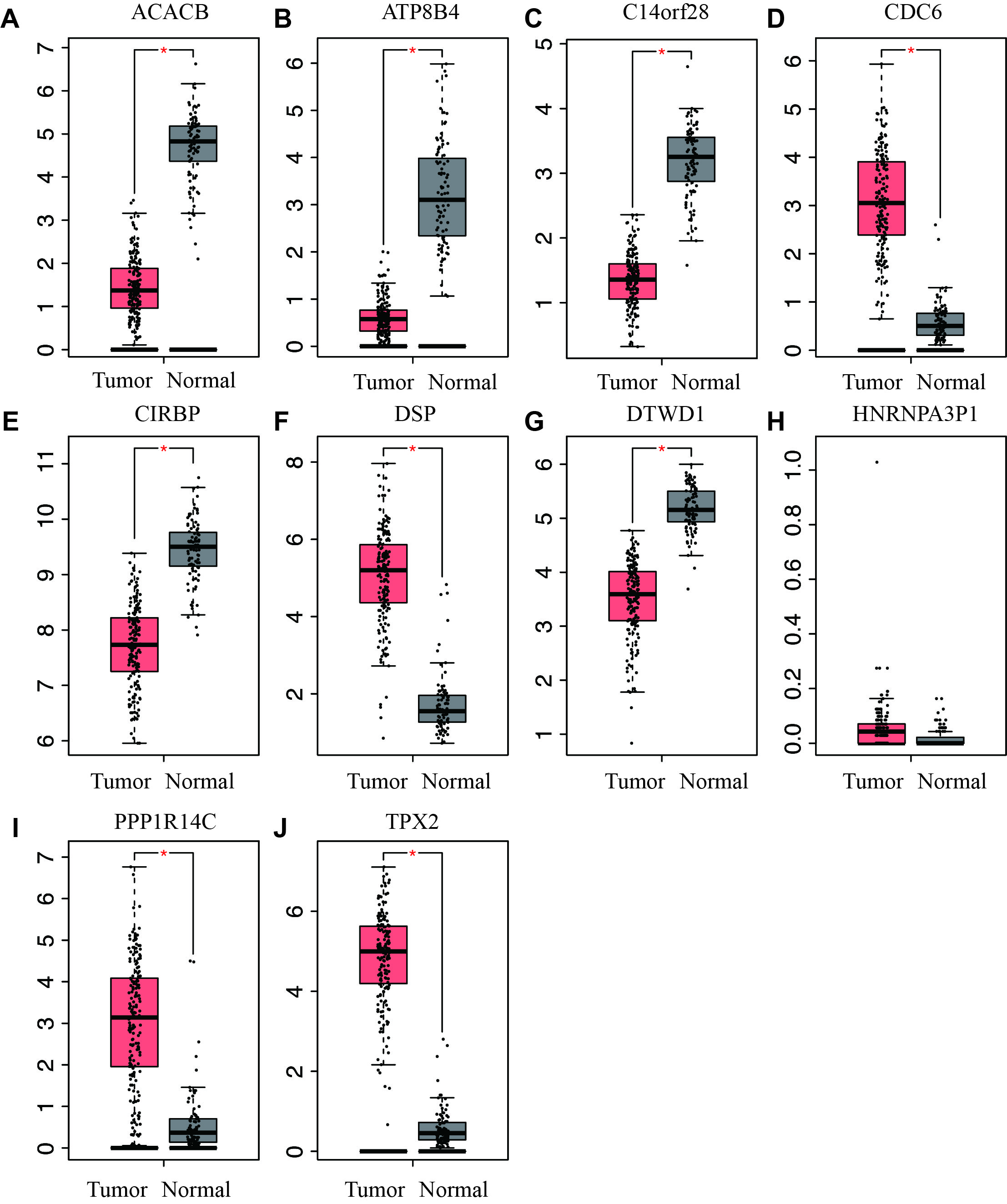

Supplement: Supplementary file 2 — Figure S2 [file CAM4-11-4641-s005.jpg]

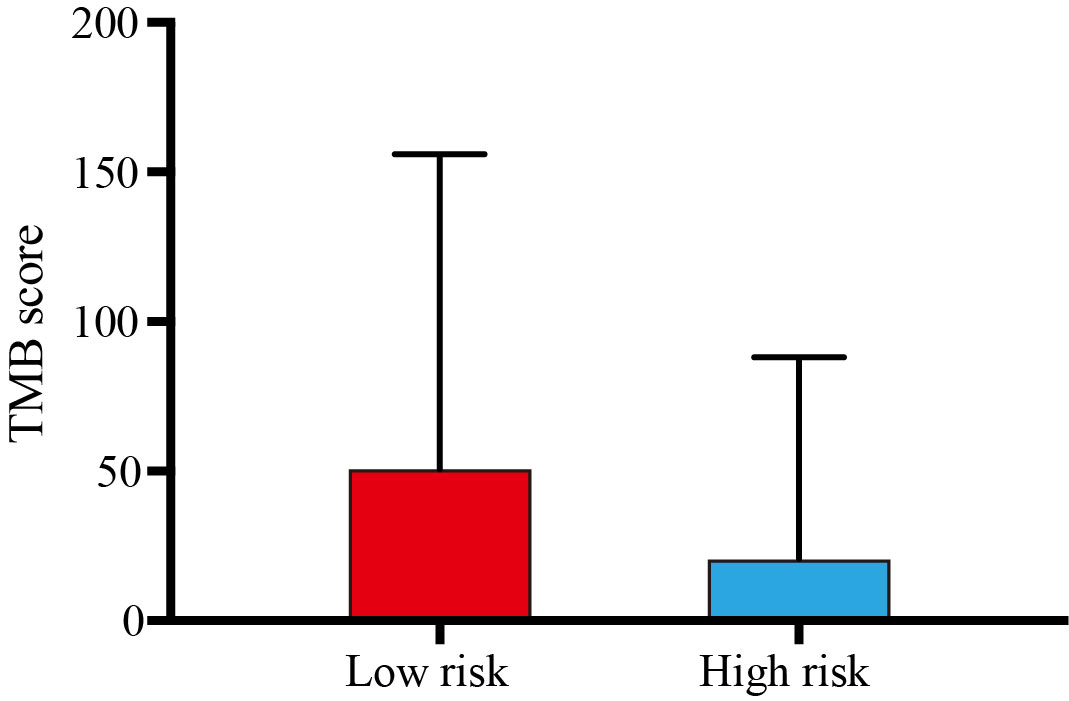

Supplement: Supplementary file 3 — Figure S3 [file CAM4-11-4641-s008.jpg]

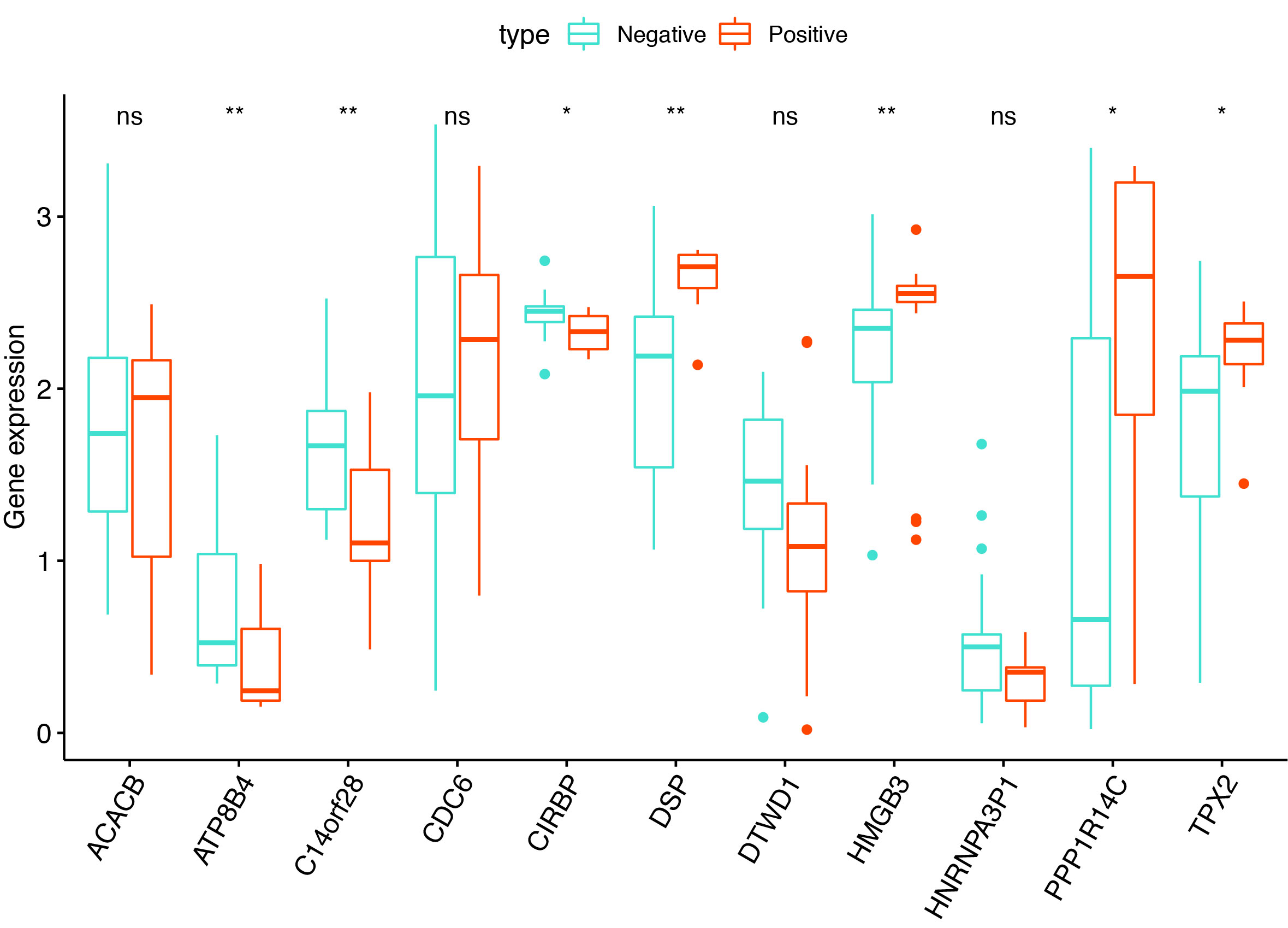

Supplement: Supplementary file 4 — Figure S4 [file CAM4-11-4641-s007.zip › cam44844-sup-0004-FigureS4A.jpg]

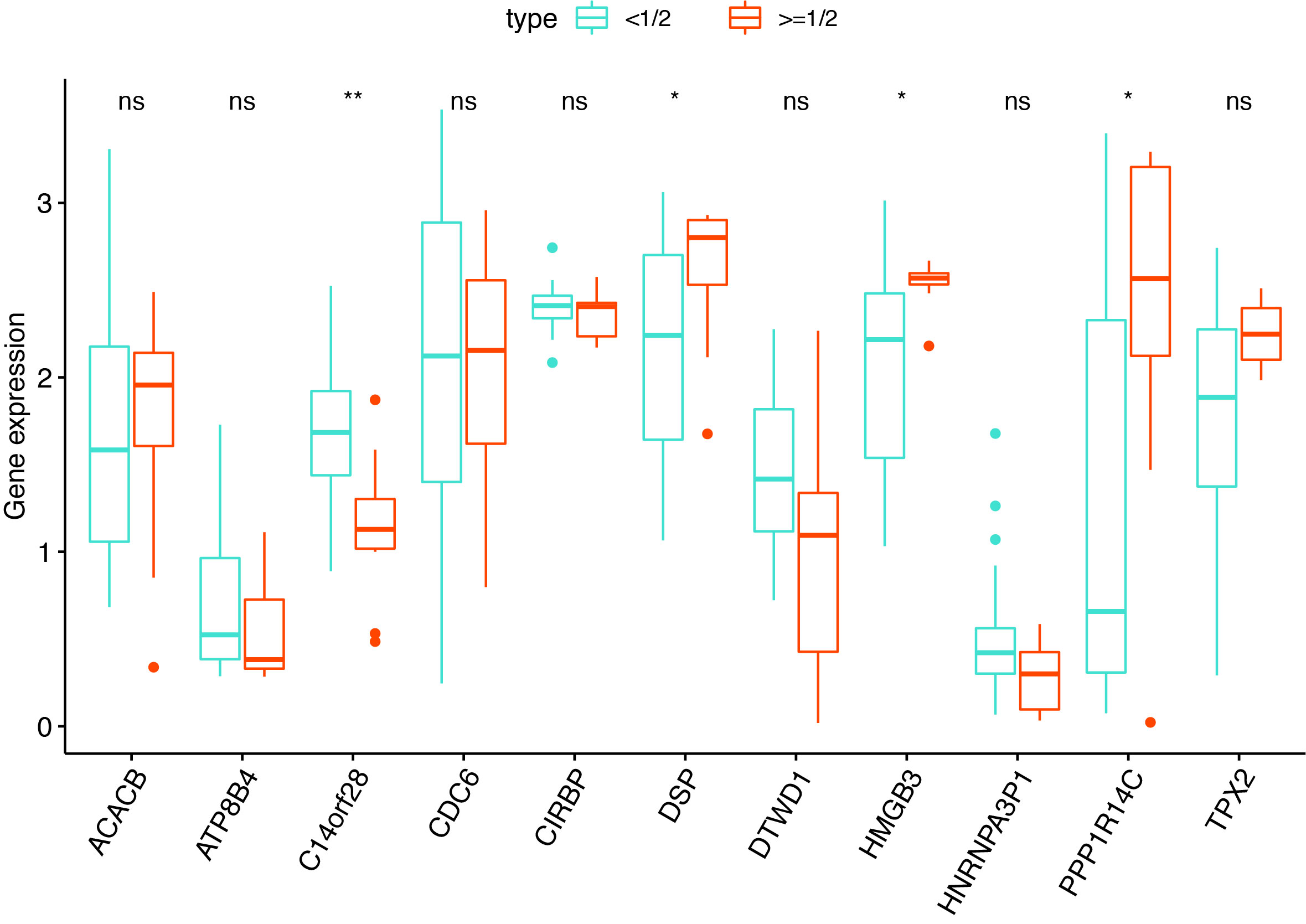

Supplement: Supplementary file 4 — Figure S4 [file CAM4-11-4641-s007.zip › cam44844-sup-0005-FigureS4B.jpg]

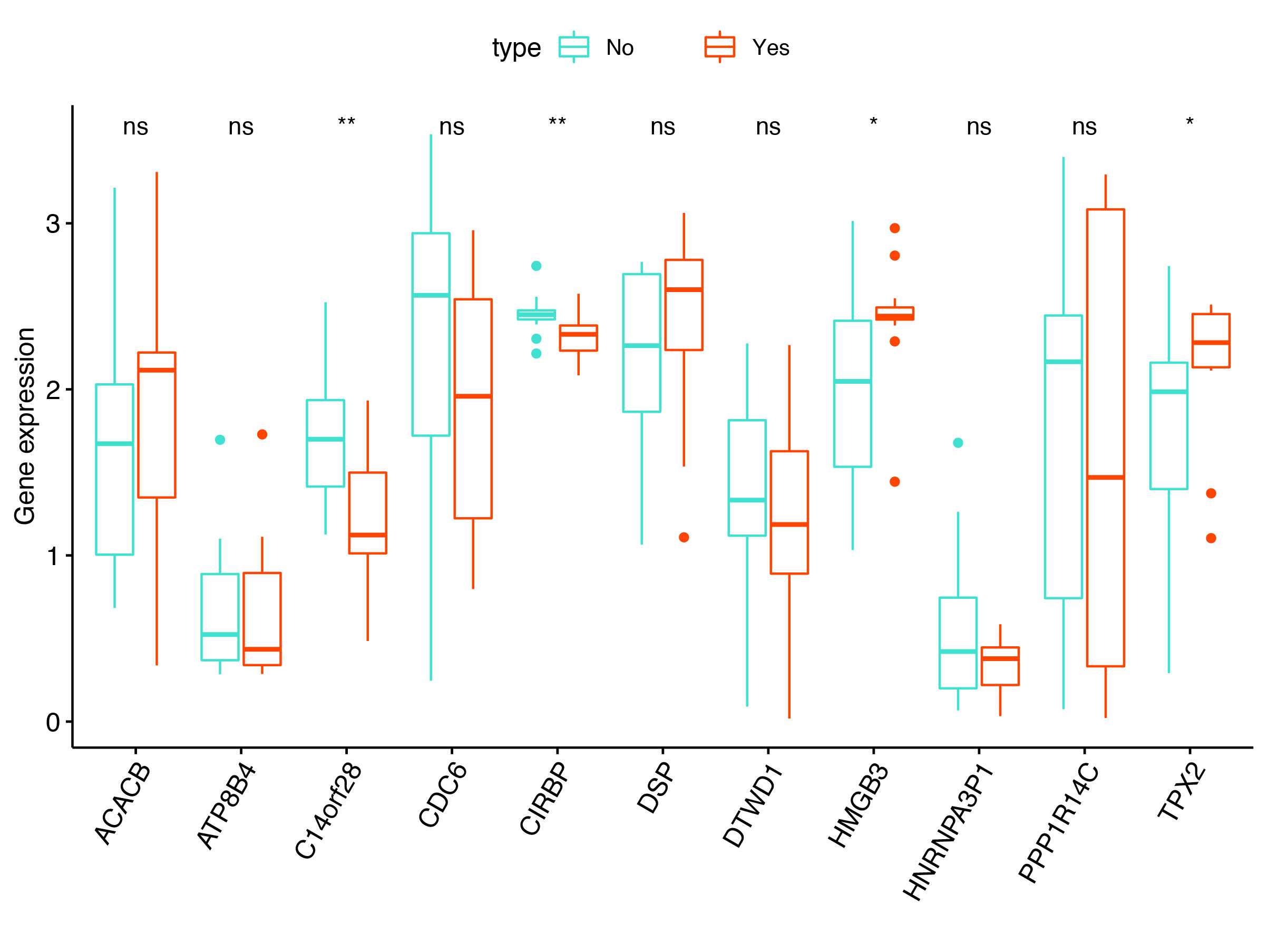

Supplement: Supplementary file 4 — Figure S4 [file CAM4-11-4641-s007.zip › cam44844-sup-0006-FigureS4C.jpg]

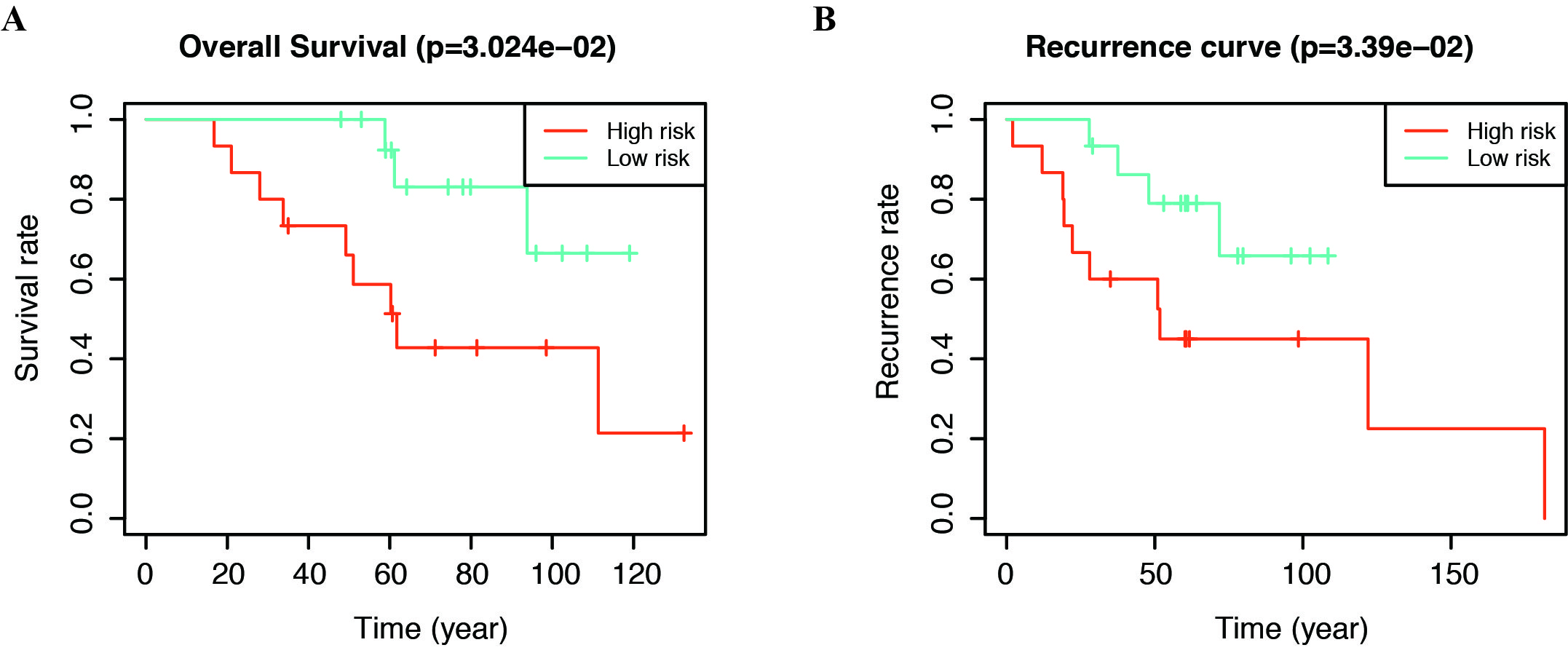

Supplement: Supplementary file 5 — Figure S5 [file CAM4-11-4641-s001.jpg]

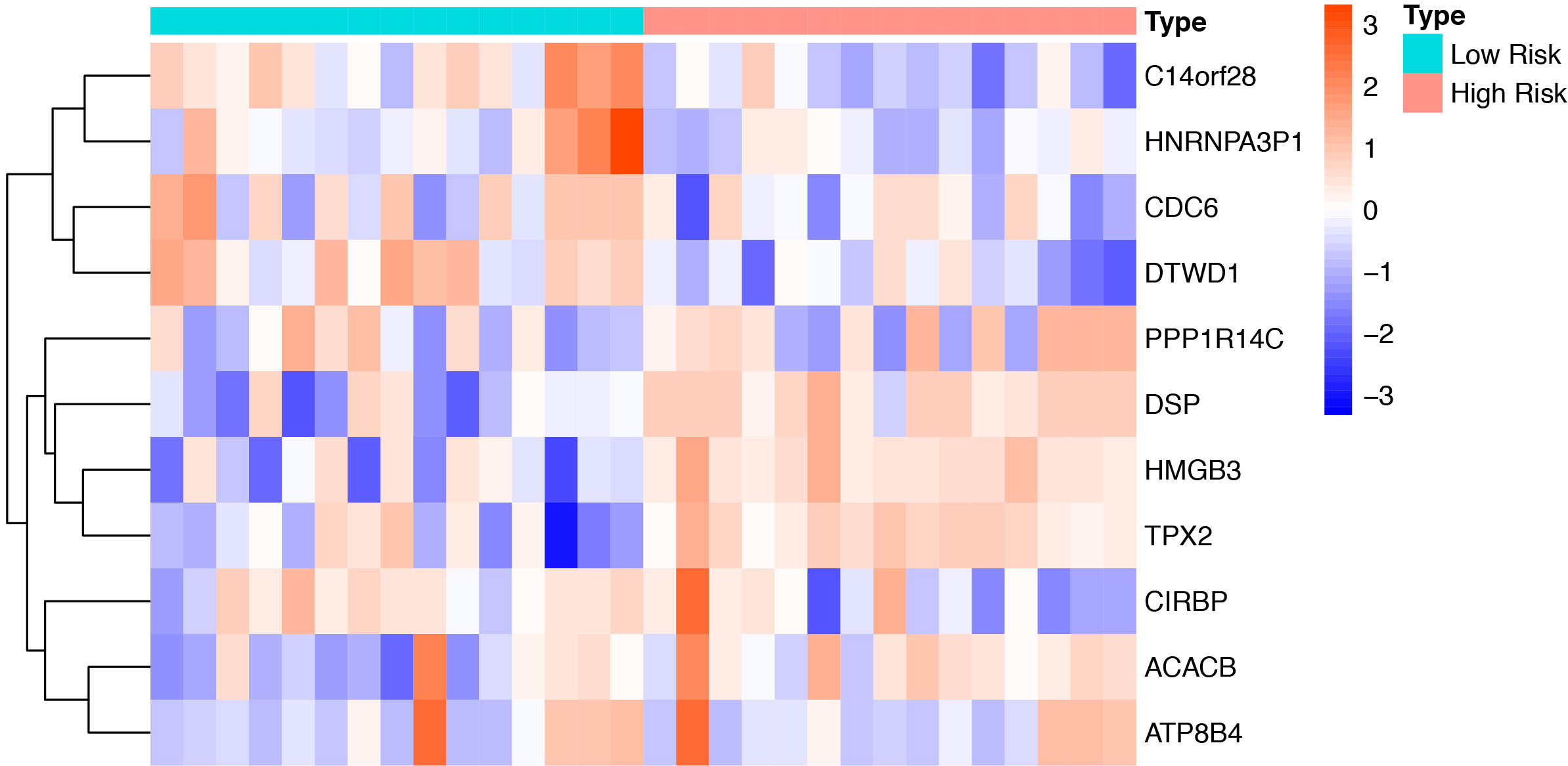

Supplement: Supplementary file 6 — Figure S6 [file CAM4-11-4641-s002.jpg]

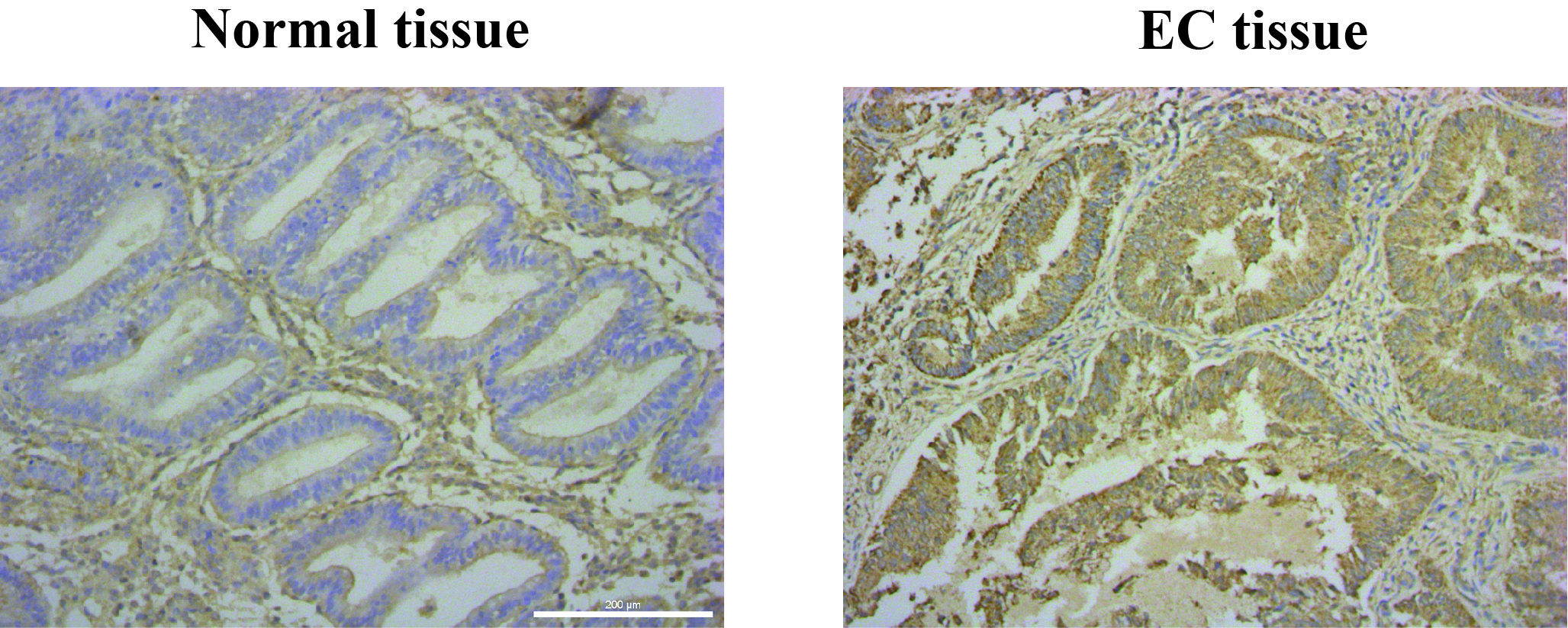

Supplement: Supplementary file 7 — Figure S7 [file CAM4-11-4641-s009.jpg]
