# Supplementary material for: Construction and validation of a prognostic model based on 11 lymph node metastasis‐related genes for overall survival in endometrial cancer
Source: Cancer Med. 2022 Jul 2;11(23):4641–55. doi: 10.1002/cam4.4844 (PMC9741985; doi:10.1002/cam4.4844)
Supplement: Supplementary file 9 — Table S2 [file CAM4-11-4641-s003.docx]

Table S2 Characteristics of patients in TCGA cohort

| Variables | Cohort |
| --- | --- |
| Total number | 515 |
| Age (year) | 64.20 ± 11.0 |
| OS (day) | 999.7 ± 854.5 |
| Living status |  |
| Alive | 423 (82.45%) |
| Death | 92 (17.55%) |
| Diabetes |  |
| No | 350 (68.75%) |
| Yes | 165 (31.25%) |
| Hypertension |  |
| No | 196 (38.22%) |
| Yes | 319 (61.78%) |
| Menopausal status |  |
| Premenopausal status | 81 (15.87%) |
| Postmenopausal status | 434 (84.13%) |
| FIGO stage |  |
| Stage I | 314 (61.30%) |
| Stage II | 51 (10.10%) |
| Stage III | 113 (22.83%) |
| Stage IV | 37 (5.77%) |
| Tumor grade |  |
| G1 | 82 (14.66%) |
| G2 | 110 (21.39%) |
| G3 | 323 (63.94%) |
| Histological type |  |
| EEA | 371 (72.36%) |
| Other types | 144 (27.64%) |
| Recurrence |  |
| No | 410 (79.57%) |
| Yes | 105 (20.43%) |
| Peritoneal cytology |  |
| Negative | 426 (82.69%) |
| Positive | 89 (17.31%) |
| LNM |  |
| Negative | 370 (72.60%) |
| Positive | 145 (27.40%) |

OS, overall survival; FIGO, The International Federation of Gynecology and Obstetrics; G, grade; EEA, endometrioid endometrial adenocarcinoma; LNM, lymph node metastasis
